# Digital Slide Assessment for Programmed Death-Ligand 1 Combined Positive Score in Head and Neck Squamous Carcinoma: Focus on Validation and Vision

**DOI:** 10.3389/frai.2021.684034

**Published:** 2021-06-04

**Authors:** Albino Eccher, Ilaria Girolami, Giancarlo Troncone, Liron Pantanowitz

**Affiliations:** ^1^ Department of Pathology and Diagnostics, University and Hospital Trust of Verona, Verona, Italy; ^2^ Division of Pathology, Central Hospital Bolzano, Bolzano, Italy; ^3^ Department of Public Health, University of Naples Federico II, Naples, Italy; ^4^ Department of Pathology and Clinical Labs, University of Michigan, Ann Arbor, MI, United States

**Keywords:** training, validation, artificial intellegence, combined positive score, digital pathology and image analysis, whole slide image, programmed death ligand 1, head and neck squamous cell cancer

## Introduction

**FIGURE 1:**
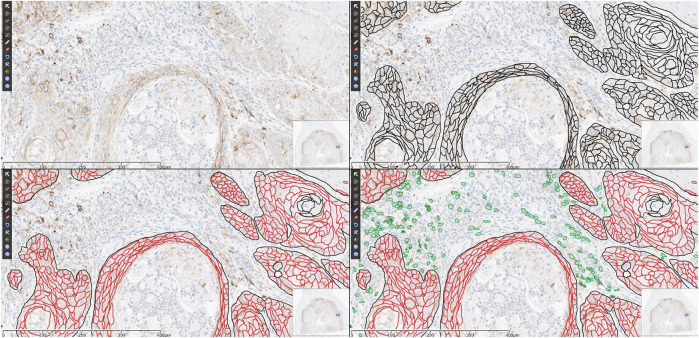
Suggested logical flow of CPS assessment. In a field at 20x magnification [panel **(A)**], the first step is to identify total viable tumoral cells, here highlighted in black [panel **(B)**], then the positively stained tumoral cells are counted [red, panel **(C)**] and finally positively stained immune cells are added to the numerator [green, panel **(D)**]. In this example with manual annotations, the CPS is (496 positive tumoral cells + 168 positive immune cells)/535 viable tumoral cells × 100 = 124, which is considered 100 for scoring purposes.

Head and neck squamous cell carcinoma (HNSCC) represents a leading cause of mortality in some countries. The 5-years overall survival has improved modestly over the past 3 decades and is still only at 50–65% despite combined surgical resection and radiotherapy and/or chemotherapy (Johnson et al., 2020). In recent years, new immunotherapy drugs inhibiting the interaction between programmed death 1 (PD-1) expressed on T-helper lymphocytes and its ligand programmed death-ligand-1 (PD-L1) expressed on cancer cells have been utilized as an effective treatment for different types of advanced cancers including HNSCC. The binding of PD1 to its ligand PD-L1 normally reduces the proliferation and activity of cytotoxic CD8 T lymphocytes against presented antigens, thus inducing self-tolerance. The introduction of immunotherapy drugs targeting the PD-1/PD-L1 axis represented a turning point in the therapy of HNSCC. PD-L1 status is assessed by immunohistochemistry (IHC) using the combined positive score (CPS). This integrated scoring system considers the expression of the PD-L1 immune checkpoint biomarker on the cell membrane of both tumor and tumor-associated inflammatory cells. The CPS assessment is often performed on small biopsy material, as candidate patients present with advanced cancer are often unfit for large surgery as are those with recurrence after adjuvant therapy. PD-L1 expression is associated with an increased objective response rate to therapy, with better responses observed when the CPS ≥ 20, as was recently shown in clinical trials investigating the efficacy of this first-line immune checkpoint inhibitor in recurrent and/or metastatic HNSCC (Burtness et al., 2019). This finding is crucial for clinicians in order for them to prescribe the best course of treatment for cancer patients. Consequently, it is to be expected that pathologists are increasingly requested to assess companion PD-L1 CPS on HNSCC specimens in order to meet the rising demand of selecting suitable patients for immunotherapy. Reliable immunohistochemical assessment of PD-L1 requires not only expertize in head and neck pathology, but also validated IHC assays and tailored training for accurate, standardized and reproducible PD-L1 scoring among pathologists (Pagni et al., 2020). Efficiency and high diagnostic quality are extremely important for PD-L1 scoring. Training of pathologists is key to achieving this goal. The CPS is more complex than the Tumor Proportion Score (TPS), as it requires specific and separate counting of tumor and immune cells that are positive for PD-L1. TPS evaluates PD-L1 expression by the ratio of stained tumor cells to the total number of viable tumor cells. As depicted in Figure 1, CPS assessment requires additional steps: first of all, the total number of viable tumor cells are counted to and represent the denominator of the formula. Then the number of positively stained tumoral cell (any convincing partial or complete linear membrane staining of that is perceived as distinct from cytoplasmic staining) added to positively stained lymphocytes and macrophages (any staining at any intensity) are counted to constitute the numerator. The obtained value is multiplied by 100. CPS can outnumber 100, but this is taken as maximal possible value. When training programs by experts are provided to teach pathologists to score PD-L1 expression, the reproducibility among these pathologist appears to be high in assessing CPS, with studies reporting an intra-class correlation coefficient (ICC) of ≥0.70 (Ruiter et al., 2020) or to be excellent at ≥ 0.90 (Crosta et al., 2021). Education sessions at multi-headed microscope have recently transitioned to using digital tools, especially since the COVID-19 pandemic in order to help minimize risk to healthcare workers and trainees (Crosta et al., 2021; Eccher et al., 2021).

## Validation

Digital pathology provides innovative tools that are being increasingly exploited to improve cancer care and discovery, which includes whole slide imaging (WSI) and image analysis (Browning et al., 2020). Primary diagnosis by WSI is defined as establishing a final pathology diagnosis solely by review of digital slides without relying on manually examining glass slides using a conventional light microscope. Validation of WSI for clinical diagnostic work is a process to demonstrate that this technology performs as expected for its intended use and environment prior to using it for patient care. This helps ensure that pathologists can make accurate diagnoses with WSI to at least the same level as they can with traditional light microscopy, and that there are no interfering artifacts or technological risks to patient safety (Pantanowitz et al., 2013). Over the last 2 decades WSI has become a robust option for rendering primary diagnoses, second opinion teleconsultation, education and training, research and quality control purposes (Fallon and Prasad, 2010; Cima et al., 2018). The key components of a digital pathology system include hardware (scanner, workstation with monitor or display devices, server), software (image management system, image viewer, image analysis tools), and network telecommunications (Weinstein et al., 2009). Widespread adoption of digital pathology systems for primary diagnosis has been limited for several reasons including the cost of such systems. Direct costs include the hardware, software and informatics infrastructure. Indirect costs are related to maintenance of the system, information technology staff support, and possible expenses for training. Studies highlight the possibility of cost savings after implementation of a digital pathology system with a break-even point projected at seven years after going fully digital (Hanna et al., 2019). Another barrier to taking on WSI for primary diagnosis is the cultural resistance experienced by pathologists. Whilst it is often recommended not to substitute components (e.g., monitors) of a regulatory cleared digital pathology system in order to maintain the approved pixel pathway (i.e., from scanner to viewer), many cases today are remotely evaluated by pathologists using their own personal workstations, laptops or mobile devices. This implies that in the real world there is limited standardization of viewing displays and network bandwidth. Indeed, this was a common finding uncovered related to the COVID-19 pandemic and working remotely (Cimadamore et al., 2020), which included multicenter training events (Eccher et al., 2021). To address need to work from home during the COVID-19 pandemic, in March 2020 the College of American Pathologists (CAP) issued “COVID-19 Remote Sign-Out Guidance” that stated pathologists “may use a non-FDA approved system as long as it has been properly validated” for primary diagnosis (Borowsky et al., 2020). This statement also has implications for remote biomarker assessment, because although several digital tools were being used for this purpose (Barnes et al., 2017; Pantanowitz et al., 2020) experience with this technology was still limited.

## Future Directions

Digital pathology plays an important role in accelerating advances in healthcare by supporting collaboration. Moreover, employing digital pathology tools such as image analysis assists with discovery and scoring of predictive biomarkers. More recently, we have witnessed the application of Artificial Intelligence (AI) with WSI for breast cancer biomarkers (Barnes et al., 2017). Hence, we anticipate that similar AI tools will soon be deployed in clinical practice for assessing PD-L1 CPS in HNSCC (Taylor et al., 2019). Indeed, in a recent review by Inge et al (Inge and Dennis, 2020), AI algorithms are already being used to investigate the role of PD-L1, providing considerable insight into its expression and heterogeneity within the tumor microenvironment (Inge and Dennis, 2020). These authors showed that application of an IHC membrane algorithm for PD-L1 evaluation provided scores that were only 85% concordant with manual scoring. This is not surprising and at the same time interesting from an AI point of view. Indeed, CPS calculation requires a series of “smart” steps which are usually done manually by a pathologist when looking at a case under the microscope and which could be replaced by an algorithm: 1) recognize only areas of infiltrating tumor, excluding normal tissue, not-invasive cancer and necrotic areas; 2) count total tumor cells in fields at 20x magnification; 3) recognize lymphocytes and macrophages, excluding other immune cell populations; 4) recognize positive staining at membrane level in tumor cells and at membrane and/or cytoplasm level in lymphocytes and macrophages, and 5) build CPS as for the formula and give numerical result. The challenges encountered by such algorithms in recognizing and discriminating immune cells from macrophages are the same that are encountered by pathologists. Hence, for now we should remain cautious about the performance of these human supervised algorithms, especially since some of these studies also relied on small sample sizes. It is foreseeable that incorporation of clinical outcome as one of the benchmarks when training algorithms could provide more clinically relevant AI tools.

In conclusion, there is an emerging need in pathology to enhance and standardize the evaluation of companion biomarkers such as PD-L1 by using validated digital pathology tools to not only remotely train pathologists to provide a reliable CPS for HNSCC, but to also leverage computer-assisted tools to more accurately do so.
